# Exploring the relationship between air quality index and lung cancer mortality in India: predictive modeling and impact assessment

**DOI:** 10.1038/s41598-023-47705-5

**Published:** 2023-11-20

**Authors:** Tamanpreet Singh, Amandeep Kaur, Sharon Kaur Katyal, Simran Kaur Walia, Geetika Dhand, Kavita Sheoran, Sachi Nandan Mohanty, M. Ijaz Khan, Fuad A. Awwad, Emad A. A. Ismail

**Affiliations:** 1 Guru Tegh Bahadur Institute of Technology, New Delhi, India; 2 Maharaja Surajmal Institute of Technology, New Delhi, India; 3 School of Computer Science and Engineering, VIT-AP University, Vijayawada, Andhra Pradesh India; 4 Department of Mechanical Engineering, Lebanese American University, Beirut, Lebanon; 5 Department of Mechanics and Engineering Science, Peking University, Beijing, 100871 China; 6 Department of Quantitative Analysis, College of Business Administration, King Saud University, P.O. Box 71115, 11587 Riyadh, Saudi Arabia

**Keywords:** Cancer, Computer science

## Abstract

The Air Quality Index (AQI) in India is steadily deteriorating, leading to a rise in the mortality rate due to Lung Cancer. This decline in air quality can be attributed to various factors such as PM 2.5, PM 10, and Ozone (O3). To establish a relationship between AQI and Lung Cancer, several predictive models including Linear Regression, KNN, Decision Tree, ANN, Random Forest Regression, and XGBoost Regression were employed to estimate pollutant levels and Air Quality Index in India. The models relied on publicly available state-wise Air Pollution Dataset. Among all the models, the XGBoost Regression displayed the highest accuracy, with pollutant level estimations reaching an accuracy range of 81% to 98% during training and testing. The second-highest accuracy range was achieved by Random Forest. The paper also explores the impact of increasing pollution levels on the rising mortality rate among lung cancer patients in India.

## Introduction

Globally India has been ranked 5th^1^ country with highest air pollution among 118 countries. In 2019, a worldwide survey^2^ was conducted that determined among 30 most polluted cities 21 cities are in India US AQI -EPA’s index of reporting air quality. The Five major pollutants are:-Ground-level ozone (O3), Particle Pollution (also known as particulate matter, including PM2.5 and PM10), Carbon Monoxide (CO), Sulphur Dioxide (SO2), Nitrogen Dioxide (NO2). In 2021, average US AQI of India is 151, according to the^1^ daily AQI Colour is red being Level of Concern Unhealthy. The average PM2.5 concentration in India is 11.6 times the WHO annual air quality guideline value. Bhiwadi, Rajasthan was most polluted city in 2021 being AQI 177^1^, Unhealthy level of concern. As of 24 Oct, 2022 Delhi’s AQI is 256, Very Unhealthy^2^. Majorly lung cancer patients are highly affected by the constant rise of particulate matter concentration in the air^2^. According to the Exploratory Data Analysis of the data provided by Kaggle^3^, Ahmedabad^2^ has the highest concentration of NO_2_, SO_2_, CO whereas Delhi with highest concentration of PM2.5, PM10 and NO_2_.

Among all the types of cancers Lung cancer is the huge cause of increasing mortality in India and is highly affected by the Air Quality Index. Lung cancer patients are vulnerable to degrading lung capacity and respiratory diseases due to the major rise in the composition of pollutants in the air. World-wide Lung cancer is the major cause of concern due to the increasing rate of death cases. According to the 2020 National Cancer Registry Programme report^4^ 1 in every 6 males and 1 in every 7 females had a cumulative risk of developing cancer of any site in the age of 0 to 74 years in India. A trend of gradual growth in Lung cancer cases and deaths have been noticed with the gradual increase in air pollution over past years. Lung cancer has become a historically dominant type of cancer in the past decade.

**Figure 1:**
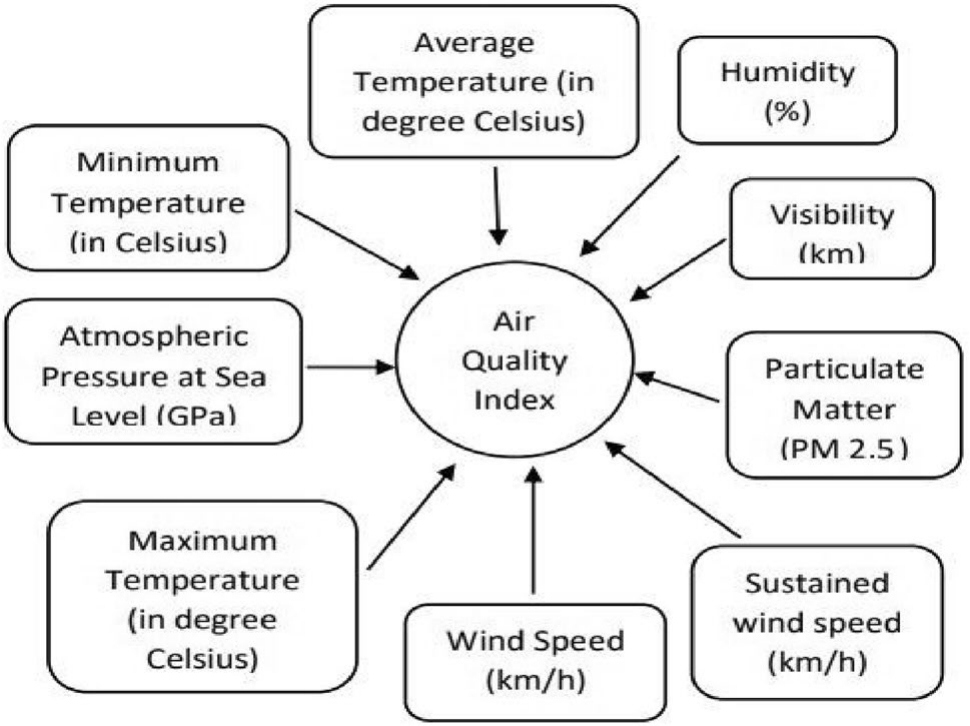
Factors affecting AQI.

The rise in particulate matter as well as the alternative pollutants depicts a concerning issue to the community due to the impurity of air. There are various features affecting the quality of air some of which are shown in Fig. 1.

Lung cancer being the most familiar and dangerous cancer can develop some interest about the interrelation among the lung cancer and pollution caused by the air.

To evaluate copious data together with analyzing patterns and trends which is not easy for person to do can be done by applying some machine learning tools which can be utilized for the anticipating of the places having greater amounts of pollution and the possibility of having lung cancer. Machine learning algorithms can figure out the patterns and trends by getting trained on given historical data for the forecasting of the future status of the quality of air based on some different factors.

The correlations between the air quality index and the lung cancer can be established by using the machine learning techniques which can gives out the desired results which can be used further for the diagnosis of the public health. Machine learning techniques further used in the given research are:

1. Linear regression: This algorithm is known as one of the simplest and well-liked machine learning algorithms. Predictive analysis has been done by using the analytical algorithm. It predicts the relationship between the continuous variables and depicts the relation between the x-axis and y-axis which are independent and dependent variables respectively. Regression having a single input variable(x) is known as the simple linear regression whereas having multiple inputs is known as multiple linear regression. It depicts the relationship between the variables by giving a sloped straight line.
  $$y \, = \, mx \, + \, c$$
2. K-nearest neighbor: KNN is termed as the easiest Machine Learning algorithms established on supervised learning presuppose the closeness between the new case/data handset the unfamiliar case into the obtainable categories which have almost resemblance. Depending upon the resemblance it collects all the accessible data and creates a new data point. This implies when new data is visualized it can be simply designated into an appropriate category. KNN is a non-parametric algorithm, which states that it would not give any conjecture on provided data. This algorithm fails to give any conjecture from the training set instantly rather saves the dataset. At classification time, it executes an operation on the latest so it is also known as lazy learner algorithm.
3. Decision tree: Decision tree is simplest algorithm to use, it is used to provide a solution to a particular problem. It starts with roots and then branches off in various solutions just like a tree. It is a part of supervised learning algorithms which is used to solve classification problems and regression problems both. It is used to create a training model which predicts the class of the target variable by learning decision. It asks questions and on the basis of the answer it splits the tree into further subtrees. On this comparison, it goes along with the branch that leads to that value and then jumps to the next node. The start of a decision tree is known as a root node, the root node then splits and the initial split is called decision nodes, they are known as decision nodes because a split is made which causes the tree to branch in two directions. The leaf node often leads to the final answer or the predicted value.
4. Artificial neural network: Artificial intelligence provides a sub field known as the artificial neural network which tries to perform different tasks and designed the way in which the brain functions. It is being organized in a way in which the neurons are interconnected having layer of networks with each and every layer of the network. It receives the input signal from the outside sources in the pattern and the vector form. there are two artificial neural network topologies feedforward and feedback.
5. Random forest regression: Random forest regression is a machine learning approach which clarifies the classification and regression problem and contains plenty of decision trees. The ‘Forest’ drawn from this technique is qualified by bagging or bootstrap aggregating. To elevate the efficiency, it uses bagging as an ensemble meta-algorithm. The conclusion of this algorithm is based upon the forecast of the decision tree by taking the mean output from different trees. It has been concluded that random forest regression is more reliable than the decision tree model and gives an adequate way of approaching missing data without hyper-parameter tuning. Random forest regression clarifies the problem of overfitting in decision trees.
6. XGboost regression: This algorithm is based on class ensemble algo which is used for prediction modelling scenarios. It is open-source library that is easily accessible by all. It provides efficient and effective implementation.

Research article on lung cancer and its relationship with the Air Quality Index (AQI) may be justified by involve machine learning (ML) as an important use case for an array of solid reasons: (1) data complexity and volume: studies involving environmental factors like AQI and health effects like lung cancer frequently require huge and complex datasets, which the use of machine learning is excellent at handling. (2) Pattern discovery: ML algorithms could uncover connects and patterns in data that could not have been seen using traditional statistical methods. When employed on data related to lung cancer and AQI, ML can reveal complicated relationships that could provide light on the subtle interplay between external factors and health. (3) Predictive modelling: based on former AQI data and other relevant variables, predictive ML models, in particular models like regression and classification algorithms, can be used to anticipate lung cancer incidence or risk. (4) Feature selection: using ML approaches, it is possible to automatically select the most important variables from a pool of candidate predictors. When it comes to lung cancer and AQI, ML can assist identify which air quality indicators are most closely associated with the condition, offering crucial information for public health initiatives. (5) Real-time analysis: models based on machine learning (ML) may be taught to evaluate air quality index (AQI) data in real-time, opening the door to the possibility of prompt interventions and warnings in places where the quality of the air is quickly declining. (6) Non-linear relationships: machine learning models may represent non-linear and complicated links among AQI and lung cancer risk, in contrary to classic statistical approaches, which frequently presume linear relationships. As a consequence, the data may be interpreted in a more subtle manner. (7) Scalability: ML techniques are beneficial for studying lung cancer in different regions and demographic groups impacted by different levels of air pollution because they can scale to accommodate enormous regions and diverse populations. (8) Continual learning: as they are exposed to new data, ML models can evolve and get better over time. The research may grow more dynamic and relevant as a result of this adaptability, which can increase its accuracy of forecasts and insights. (9) Improved decision support: ML may offer policymakers and healthcare professionals significant decision support tools. (10) Innovation and progress: incorporating machine learning (ML) in your research advances the methodology of science. In conclusion, using machine learning as a use case in study demonstrates the importance and practicality of cutting-edge techniques in solving urgent public health concerns like lung cancer in connection to air quality.

## Background and motivation

The changing demographics of lung cancer with each passing year has shown that Lung cancer has develop into the prominent cause of deaths due to cancer not only in India but worldwide. In India, according to the previous year statistics reports lung cancer is responsible for 5.9%^5^ among all types of cancer. Along with this 8.1%^5^ of all cancer deaths are due to lung cancer.

Lung cancer is also termed as lung carcinoma. It basically causes uncontrollable growth of cells in the lung tissue. The two types of lung cancer namely Small Cell Lung Cancer (SCLC) and Non-Small-Cell Lung Cancer (NSCLC). Majorly people are affected by lung cancer due to smoking but nowadays non-smokers are also highly prone to lung cancer because of the air pollution. According to the statistical reports of 2019 the concentration of PM2.5 in air was so high that it was equivalent to smoking 26 cigarettes^5^. There are two major types of SCLC—[A] Sc—Squamous cell carcinoma [B] ADC—adeno- carcinoma.

The cancer that origins in the lungs’ cells, commonly present either in the bronchi’s lining (air carrying tubes for in and out) or inside the petite air containing sacs named as alveoli is recognized as lung cancer. Moreover, it usually diagnosed at a higher stage when curing opportunities are bounded that’s why it is the most typical and lethal tumor. According to the demographics there are the majority of males or females that got affected by the disease are smokers and majorly males constitute to the highest ratio of lung cancer patients. Another fact that should be taken seriously is the ratio of doctors in India according to the demographics, there is only 1 doctor per 1456 people whereas Doctor- population Ratio as recommended by WHO is 1 doctor per 1000 people^5^. Hence, the need to subject this issue of rising Air quality Index with every day and rising mortality rate by lung cancer is required.

The increasing risk factors that are leading towards this high rate of mortality and found it to be a major issue of concern by the government of India. The government of India should take some preventive measures to ensure the control of air pollution and also create some awareness about lung cancer to prevent the gradual rise of lung cancer patients.

Assorted aspects that can influence an individual’s uncertainty of growing lung cancer are indicated as adjustable and non-adjustable.

### Non-adjustable

1. Age—the possibility on diagnosed with a lung cancer is higher for elderly people as it grows with age.
2. Family’s past records and genetics—a family representative having a past record of lung tumor can increases the risk and ancestral mutations can be linked with a greater possibility of having a lung cancer.

### Adjustable

1. Taking substances containing tobacco such as cigarettes, cigars and some chemicals namely carcinogens such as asbestos, arsenic, and diesel exhaust during occupational Exposure like Working in places namely mining, construction etc. can become a primary cause for having lung cancer.
2. Sitting frequently around smokers regularly can lead to the exposure of having a lung cancer for non-smokers (passive smokers).
3. Persistent exposure to greater levels of air contamination mainly in metropolitan areas has been bound with a greater risk of having a lung cancer.

Some of the effects of lung cancer are Constant cough, coughing up blood, chest pain, indescribable loss in weight, Effects of medical care such as surgery, chemotherapy immunotherapy can cause alopecia, changes in food desires and tiredness.

One of the major problems that India faces today is air pollution. Air pollution in India is majorly caused by the pollution coming from the various factories, industries and vehicles in urban areas. In rural areas it can be caused by large scale burning of crops in fields. Air pollution can also be caused my burring of diesel, petrol, biomass coal and other fossil fuels etc. High level of pollutants and impure matter cause a great threat to the health of the population in fact air pollution has been linked to lung cancer which means that high level of air population can cause lung cancer which is the reason of great concern as lung cancer is one of the deadliest types of cancer prone to human beings. According to world health organization^6^ 22 out of 30 most polluted cities are located in India which in turn results in approximately 1.5 million deaths per year. There are almost 67,000 cases^6^ which are registered per year of lung cancer which is caused my high level of air pollution.

In this paper various machine learning techniques are used which is an efficient tool, to analyse a huge volume of data and then help to identify the places which are most at risk of air pollution. By finding out these places it would be easier for the public health strategies to reduce pollution in that particular area that would bring down the total percentage of cases of lung cancer. Machine learning helps the researchers to do a close analysis of the relationship between air pollution and lung cancer and then make models which is used to predict the air quality and then helps in finding the area’s most at risk of air pollution which reduces lung cancer. This project will contribute to counter the increasing AQI hence also creating awareness about the harmful effects of AQI for Lung cancer patients and many other patients suffering from cancer, lung diseases or respiratory diseases. Since air pollutants majorly affect not only Lung cancer patients but also affect the lung capacity of everyone leading to respiratory diseases such as asthma, chronic bronchitis, COPD and more.

## Literature review

This section of the research comparises of a brief review of past papers / research regulating the issue and severe consequenses of rising AQI and lung cancer all over. Kalaivani et al.^7^ stated about the detection system made by the use of deep learning techniques. The dataset of Computed Tomography (CT) images was taken up for the detection purposes. Further for the classification of the lung images dataset the images are classified as normal or malignant. A densely connected convolution neural network basically a DenseNet layer made for the classification and image detection purposes. Total 201 images were used and the train test split of 85–15 was encountered at the time of model configuration. Deep Learning is used since it provides better feature engineering than Machine Learning on its own. The accuracy of 90.85% was obtained by the proposed model.

Kumar et al.^8^ stated the quality of air being highly dependent on the number of pollutants affecting the health of humans. The pollutant release from industries, vehicles being the major cause of air pollution in India the dataset comprises 23 Indian cities of the past six years. An exploratory data analysis was done after the feature scaling of the dataset to provide more visualised experimental results that can be concluded by the given data. The dataset has been resampled and further different techniques are used for air quality prediction model such as KNN, Gaussian Naïve Bayes, SVM RF and XGboost. The best accuracy provided was 91% by XGboost Model.

Wei Soh et al.^9^ proposed a deep learning approach to use deep learning methods to forecast air quality for 2 days. The paper suggested the use of multiple neural networks with a combination of ANN, CNN and LSTM. Further providing the air quality predictive system over meteorological dataset. The proposed model observes higher results in specified regions giving the best accuracy. The visualization techniques are used for calculating the RMSE for both the training and testing dataset. The best performance was observed in Taiwan and Beijing.

Subramaniam et al.^10^ stated the effect of air pollution on human beings by exploring the application of AI in predicting air pollution. in this paper author used many technologies such as decision tree, machine learning and neural networks in forecasting the air pollution and its effect on human health^11^. it is evident that these technologies improve the accuracy of predicting air pollution which would help in reducing it effects was something which was argued by the author. several different approaches such as machine learning algorithms, chemical transport models and statistical models which also help in predicting air pollution. the author states the limitations of current situation and future scope for predicting air pollution as he concludes the paper. this paper highlights an important overview of the need of AI technologies and their potential in predicting air pollution and why it’s important to continue the research in this particular field.

Previous researchers discussed solely about the Air pollution describing the levels of AQI and the gradual increase in the Air pollution all over India using the political maps of India showcasing the different levels of Air Quality Index with tables of AQI colour levels also mentioning the level of concern with respect to range provided. The data for such papers is made available at IQAIR website where one can check for worldwide air quality index at any time. These research papers are further discussed in Table 1.

**Table 1:** List of papers reviewed for AQI parameter.

S. no.	Paper, author name	Dataset	Algorithm	Main concerns of paper
1	Deep learning based lung cancer detection and classification by N. Kalaivani et al.^7^	Dataset of 201 lung images (CT images)	Deep learning, DenseNet, Image Processing, Convolution Neural Network	Lung cancer
2	Air pollution prediction with machine learning: a case study of Indian cities by K. Kumar et al.^8^	Air pollution dataset have been extracted from Central Pollution Control Board (CPCB)	Machine Learning, KNN, Gaussian Naïve Bayes, SVM, RF, XGboost	Air pollution prediction in Indian cities
3	Adaptive deep learning based air quality predic tion model using the most relevant spatial temporal relations by P. Wei Soh et al.^9^	Meteorology data	Deep learning, Multiple Neural Networks including ANN, CNN, LSTM	Air quality prediction
4	Artificial intelligence technologies for forecasting air pollution and human health: a narrative review by S. Subramaniam et al.^10^	Air pollutants data	Hybrid machine learning models	Impact of air pollution on human health
5	Air quality effects on health and approaches for its assessment through microfluidic chips by F. Schulze et al.^12^	PM2.5 data	Machine learning and AI	PM2.5 air quality affecting lungs
6	A study on prediction of lung cancer using machine learning algorithms by A. Gupta et al. ^13^	Lung cancer image dataset	Machine learning, SVM, random forest, KNN	Lung cancer
7	Lung cancer prediction from text datasets using machine learning by A. Kumar et al.^14^	Text dataset	Machine Learning, SVM Classifier, KNN, Naïve Bayes, SMOTE methods	Lung cancer
8	Air quality prediction using classification techniques by Sumathi et al.^15^	Data on pollutant level is extracted by environmental monitoring models	Machine Learning, Deep Learning Techniques	Air quality prediction
9	Using machine learning to predict air quality index in New Delhi by S. Bhattacharya et al.^16^	Archive pollution data by CPCB and US Embassy in New Delhi	Machine Learning, SVR, RBF (Radial Basis Function)	Air quality prediction
10	Lung cancer in India D. Behera et al.^17^	World-wide lung cancer data	–	Lung cancer its correlation with AQI and its effects
11	Lung cancer risk prediction with machine learning models by E. Dritsas et al.^18^	Lung cancer dataset available on Kaggle	Machine Learning, SVM, ANN, NB, DT, KNN	Lung cancer risk predictive system
12	Lung cancer prediction and classification based on correlation selection method using machine learning techniques by D. Mustafa et al.^19^	UCI datasets for lung cancer patients	Machine Learning, SVM, KNN, and CNN	Lung cancer predictive system
13	Lung cancer classification and prediction using machine learning and image processing by S. Nageswaram et al.^20^	83 CT scans photo data was obtained by 70 patients	Machine Learning, ANN, K means, KNN and Random Forest	Lung cancer classifications and predictions

The Table 1 discusses about the Dataset and algorithms used by the researchers in the explained papers. Hence, providing a comprehensive detailed analysis on the Literature review provided in the paper.

Schulze et al.^12^ stated the effects of air pollution on health and how microfluidic chips is used to fix these effects. An overview of particulate matter, nitrogen dioxide and Oxone which are found in air and their harmful effects on health such as cardiovascular and respiratory diseases was given by the author, he further discusses the problems involved with measuring the air quality which includes the need for real-time monitoring data. The author highlights use of microfluidic chips which are a solution for fixing the quality of air, they are basically small tools which control fluids of small volume that enables the air pollutant measurement concentrations in real team; there are various kinds of microfluidic chips which are developed over time to monitor the quality of air and which measure particulate matter. The author concludes the paper by highlighting the fact that there is a need to continue research so that the accuracy of microfluidic chips can be improved and their application is expanded to a larger range of pollutants.

Gupta et al.^13^ This paper stated that for predicting the presence of lung cancer the usage of machine learning algorithm is very important. In this paper various algorithms of machine learning such as support vector machines, decision trees, random forests are used in predicting lung cancer. The author talks about the conventional ways of diagnosing lung cancer and how important more effective methods are for early detection of lung cancer. The dataset that is used by the author included clinical features, images of CT scan and demographics. The author highlights how important the use of feature selection is in improving the performance of the machine learning models. The paper is concluded by discussing the limitations of current methods and the future directions of this study. The author describes the importance of clinical validation and the need of large and more diverse dataset to enhance the performance of machine learning algorithms.

Kumar et al.^14^ In this paper machine learning algorithms were used using text datasets for predicting the presence of lung cancer which included logistic regression, naive bayes and decision trees. The author describes how lung cancer is diagnosed and treated using current methods and then gives an overview of dataset used in this paper that is pathology reports and text data from medical records of the patient. The accuracy and effectiveness of machine learning algorithms from text datasets for predicting lung cancer was presented by the author, he also highlighted how salient feature selection is in improvising the performance of lung cancer. The paper is concluded by stating that there is a need for more diverse and larger amount of dataset for improving the further performance and importance of clinical validation and how critical the synergy between medical professionals and computer scientists is.

Sumathi et al.^15^ has mentioned various techniques of air quality prediction. The authors explained about the detailed effect of air quality on the health of people and the environment. The dataset used in the paper is extracted from various monitoring stations. Classification techniques are used such as KNN, Decision Tree and SVM. The proposed approach predominantly discusses about the feature selection process to improve the results provided by the algorithms. Also, the ensemble methods and deep learning approach is used for optimized performance of the selected models. Hence, providing the conclusion that deep learning models are more reliable than machine learning models. The authors have also expressed the requirement of more comprehensive dataset to provide better and more accurate results.

Bhattacharya et al.^16^ talks about the air quality predictive models for AQI prediction in New Delhi. The paper has provided a detailed performance analysis of the various machine learning algorithms used. The data used in the paper was extracted by the different monitoring stations and meteorological data for air quality. The paper majorly focuses on the importance of air quality prediction for environment and healthcare services of Delhi to be precise due to the major degradation of air quality in past few years. The authors further mentioned the results and the conclusion provided by the models used in the proposed approach that is Decision Tress, Random Forests and Artificial Neural Networks also the use of hybrid models have led to more optimized predictive models. The best accuracy provided was 93.4%.

Further the brief detailing of these papers is provided in Table 2. This table provides a brief of all the papers mentioned in the Literature Review provided in this paper.

**Table 2:** A brief descriptive table.

S. no.	Paper, author name	Description	Performance parameters	Results
1	Deep learning based lung cancer detection and classification by N. Kalaivani et al.^7^	CNN model is designed to train and test the scaled image dataset. Further these trained images are moved on to the classification part to classify normal and malignant image separately	–	Accuracy of 90.85%
2	Air pollution prediction with machine learning: a case study of Indian cities by K. Kumar et al.^8^	The proposed approach extracts the dataset and further pre-process the data the key features are se- lected by the correlation analysis among them. Hence the exploratory data analysis was done and the models were made to calculate the performance parameters	R2, RMSE, RMSLE, MAE, Precision, Recall, F1-Score	Best accuracy of 91% by XGboost
3	Adaptive deep learning based air quality prediction model using the most relevant spatial temporal relations by P. Wei Soh et al.^9^	The proposed approach designed to detect air pollution sources including domestic and transboundary by using various combinational neural networks and further calculating the average train and test RMSE by visualizations	RMSE	
4	Artificial intelligence technologies for forecasting air pollution and human health: a narrative review by S. Subramaniam et al.^10^	The proposed approach discusses about the forecasting of air pollution using machine learning techniques and artificial intelligence. The major focus is on the chronic diseases due to increased air pollutants and degraded climatic conditions. Hybrid AI models provided better performance according to the researchers	R2, RMSE, MAE, MAPE	
5	Air quality effects on health and approaches for its assessment through microfluidic chips by F. Schulze et al.^12^	The proposed research discusses about the adverse effects of PM2.5. These particulate matters enter the human body and causes chronic diseases due to its small size its highly affecting the lungs of humans. Further organ on chips technology is used to study the air pollution and also the results help in making advancement in pharmaceutical drugs		Theoretical discussions and conclusion predicted on the previous researches
6	A study on prediction of lung cancer using machine learning algorithms by A. Gupta et al.^13^	Lung cancer being a major concern nowadays has led to high mortality rate due to cancer. Hence a predictive system is required for the same. The proposed theory provides the early detection and identification system for lung cancer by using the image dataset. Techniques used in the predictive model are SVM, KNN and random forest	Precision, Recall, F1 Score, Accuracy, Support	84.2% by random forest
7	Lung cancer prediction from text datasets using machine learning by A. Kumar et al.^14^	The research paper focuses on the methods to detect lung cancer using machine learning models to re- duce the time wasted in the detection purposes using SVM classifier and further comparing it with the KNN and Naïve Bayes algorithms	Precision, Recall, F1 Score, Accuracy	98.8% accuracy
8	Air quality prediction using classification techniques by Sumathi et al.^15^	The paper proposes the forecasting of air quality prediction models using machine learning or deep learning techniques and further making a comparison of which technique is better in terms of providing better and accurate air quality fore casting	Comparative in machine learning and deep learning techniques	The research suggests that the deep learning methods are more reliable for quality prediction of air pollutants
9	Using machine learning to predict air quality index in New Delhi by S. Bhattacharya et al.^16^	The paper studies a new approach rather than the traditional ap- proaches by using SVR model for determining the present air pollutants and the AQI. Dataset contain- ing various pollutants affecting the air quality for New Delhi. The best results are obtained by the RBF-SVR model	MAE, RMSE, R squared, Accuracy	93.4% accuracy by the proposed model
10	Lung cancer in India D. Behera et al.^17^	This research focuses on the theoretical aspect of lung cancer in In- dia considering the aspects i.e., effects of smoking on lung cancer, genetics, and lung cancer relationship, diet affecting lung cancer and the air pollutants affection lung cancer patients	–	Trends in lung cancer due to different factors in India
11	Lung cancer risk prediction with machine learning models by E. Dritsas et al.^18^	ML models are used to determine high risk in individuals for acquiring lung cancer and thus it helps in conquering long term complications. Various performance parameters were measured by visualization techniques	AUC, F-Measure, Precision, Recall, Accuracy	97.1% accuracy by RotF
12	Lung cancer prediction and classification based on correlation selection method using machine learning techniques by D. Mustafa et al.^19^	The paper works on the classification results of three selected ma- chine learning classifiers for the UCI patient dataset. The major use of WEKA Tool has been the highlight of the paper to predict the accuracy and other performance parameters of the given data	Confusion Matrix, F-measure, Recall, Precision, ROC curve	95.56% accuracy by SVM
13	Lung cancer classification and prediction using machine learning and image processing by S. Nageswaram et al.^20^	This research paper consists of a self-made image dataset. A major indication of geometric mean filter is subjected to be used for image pre-processing leading to increased image quality	Accuracy, Sensitivity, Specificity	ANN model provides the best result

Behera et al.^17^ presented an overall report of the load and administration of lung cancer in India. It mainly focuses on the expanding incidence as well as the fatality rates and the objections and convenience for the enhancement for the medication of the illness. The aspect of tobacco usage, uncleaned environment and the genetic aspects and the progression have been discussed by the author. They also concluded the initial disclosure of the cancer and to cut down the use of tobacco and pollution. The need of extra extensive and authentic data on the load handling and the combined passage for the avoidance, diagnosis and the prescription for the enhancement of patient’s health and to decrease the burden.

Dritsas et al.^18^ states the prediction by giving the elaborated study about the execution of discovering the lung cancer by performing some machine learning algorithms. The analysis of the cancer risk by focusing the factors of public health and climate. The dataset contains population based clinical, lifestyle and demographic data which gives the efficiency by using the feature selection through some of the hybrid models. The various machine learning models used for the analysis are the decision tree, support vector machine and the logistic regression. This evaluation shows the strength and the certainty of the algorithms by presenting the effect of feature selection and data pre-processing which enhances the model’s accuracy. It also derives the drawbacks and the coming guidelines of the study. The urgency of getting more extensive data carrying some extra features and the concern of the genetic aspects into the prediction of lung cancer to achieve stable forecasting.

Mustafa et al.^19^ examines the usability of some machine learning algorithms for the classification and forecasting of the lung cancer into various stages. It gives out the relevance of the cancer prediction for civil health by drawing the etiology and the danger aspects. The dataset used constitutes of clinical and histopathological information from victims recognised for lung cancer. The classification of the disease into the various stages is determined by using the hybrid models and some extra complicated algorithms by highlighting the need of feature selection as well as the data pre-processing which increments the results. The drawbacks, further guidance and the demand of dataset including incorporating environmental and genetic aspects for the reliable forecasting have been discussed by the author. It gives out the beneficial contribution for the improvement of the public health by enabling prior exposure and approach for the treatment of the patients.

Nageswaram et al.^20^ states the importance of techniques such as image processing and machine learning in predicting lung cancer. performance of machine learning algorithms and classification of the disease based on CT images into different stages is stated into this paper in detail. an overview of the need of lung cancer prediction with main focus on etiology and risk factors of lung cancer in provided by the author. the dataset which is used in this paper includes CT images of patients diagnosed with lung cancer. the effectiveness of machine learning analysis and other algorithms which predict lung cancer based on CT images by classifying disease into various stages. the need of feature selection and image processing is discussed in this paper which improves the efficiency of the model. while concluding the paper the author discusses limitations of the current scenario and future scopes of the study, he also states that there was a need of more comprising dataset which would have included more risk factors and features of lung cancer.

### Comparative analysis

Critical facets of technology's effects on health are covered in two different research papers.

In Kalaivani et al.'s study, main focus is on healthcare. (CNNs) and (RNNs) may be used in the analysis of medical imaging data for the early recognition and categorization of lung cancer. Due to the fact that early diagnosis considerably improves patient outcomes, this is of utmost clinical significance. The authors are likely to have performed significant data preliminary processing, involving image enhancement and feature extraction, they would have tackled the class imbalance problem common in medical datasets using techniques such as oversampling or weighted loss functions. In contrast, the research carried out by Subramaniam et al. combines the disciplines of environmental science and the health of the public. Their narrative research looks into how artificial intelligence (AI) is utilised to estimate air pollution levels and assess how it affects people's health. The approach includes an in-depth examination of the body of research, highlighting several AI techniques. The assessment likely highlights the capacity of AI to offer actual time environmental information and its implications for accurate choices regarding public health. In conclusion, these two studies serve as excellent instances for the different ways that technology is used in environmental science and healthcare. While Subramaniam et al.'s research explores how AI could assist in reducing the health risks associated with air pollution, Kalaivani et al.'s work employs deep learning for lung cancer evaluation to tackle a key medical issue. Both studies highlight how technology may be used to tackle necessary health issues and provide useful data within the fields they work in.

## Dataset used

The dataset is web scrapped from en.tutiempo.net website for 2013 to 2015 and also files have been downloaded month wise. This data contains hourly measurements of AQI^3^.

The data has been collected in different excel sheets and html sheets. Since some of this has been downloaded and some has been web scrapped from the website providing all the climate data for every country month wise.

The data contained different entries such as temperature, humidity, wind speed, precipitation, and rainfall. Further we have cleaned the manually combined data and performed the analysis on the cleaned dataset to get the desired results.

In machine learning, correcting the imbalance of classes in a dataset is a vital phase, especially when solving binary categorization issues like detecting lung cancer. The writers of your research article could have tackled the issue of class imbalance in the following ways:

1. Resampling methods: To balance the dataset, the authors may have used resampling processes. This involves either randomly including instances to the majority class (non-cancer cases) to under sample the minority class (lung cancer patients in the study) or introducing instances to the majority class (cancer cases) to overestimate it. As a result, the distribution of classes is equalised, reducing the probability that the model would be prejudiced in favour of the dominant class.
2. Weighted loss functions: In instead of resampling, the authors may have provided the classes in the machine learning algorithm varied weights in the loss function. Giving the minority class more importance pushes the model to focus on it more during training, which lowers the bias towards the dominant class.
3. Ensemble methods: Ensemble methods like Gradient Boosting or Random Forests may effectively tackle class imbalance. These algorithms gather the predictions from several models after building them. In order to organically resolve imbalance, they often give each classifier a varied weight according on the way they performed in every class.
4. Evaluation measures: The authors likely utilised assessments that are resistant to class imbalance for the purpose to assess model performance. Area under the Precision-Recall curve (AUC-PR), F1-score, confusion matrices, and the region of the Receiver Operating Characteristic curve (AUC-ROC) are popular options.

## Data pre-processing

In the proposed model the prediction and EDA process is started by importing various libraries such as—(1) NumPy, (2) Pandas, (3) Matplotlib, (4) Seaborn, (5) Io, (6) Sklearn.

Data cleaning has been done by dropping null values found in the dataset. So, the cleaned data consisted of the following factors—T stands for average temperature (degree Celsius), TM stands for maximum temperature (degree Celsius), Tm stands for minimum temperature (degree Celsius), SLP stands for Atmospheric Pressure at Sea Level (GPa), H stands for average relative humidity (%), VV stands for average visibility (km), V stands for average wind speed (km/h), VM stands for maximum sustained wind speed (km/h), PM2.5 stands for Particulate Matter.

Now this data is the cleaned data with no null values and can be used to perform feature engineering to apply different machine learning models. Feature engineering is used to create a predictive model using machine learning using the PM2.5 variable since it the key variable that is majorly affecting the lung cancer patients. PM2.5 saturation is increasing with each day as seen in the dataset the graph is used to represent the PM2.5 density over these years.

Data cleaning—the process of data cleaning started by identifying the null values in the dataset. Then the null values were dropped by the drop function. The dependent and independent features were declared namely X and y using the iloc function to be used as training and testing of the models. The training and testing data will be used as a 70–30 split from the original dataset. The correlation matrix is formulated to find the relations among the elements used in the dataset. Using these correlations, a heatmap is to be plotted. The model fitting is done using regressors. The cleaned data is used to find the increase in PM2.5 using different regression models at different rates of efficiency. The best efficiency is considered the final result of the experimental theory.

### Computational complexity and the experimental environment

The test setup, including the software and hardware environment used for the machine learning research, is explained in this section. We also go with the computational complexity related to the various machine learning methods used in this study.

#### Environment for hardware and software

On a computer cluster with strong GPUs and CPUs, the experiments were carried out. These are the main hardware environment requirements:

#### Data preprocessing

Performed many kinds of data getting ready operations, such as data normalisation, feature engineering, and handling missing values, before to training machine learning models. Python's pandas and NumPy libraries were used to complete these getting ready tasks.

#### Algorithms for machine learning

Took several types of machine learning algorithms into action and evaluated them, including but not limited to:

- Logistic regression: a basic approach that is simple but effective.
- Random forest: a robust combination method known for its capacity to handle complicated data.
- Gradient boosting: we made use of XGBoost and LightGBM, two gradient boosting methods that work well with unbalanced datasets.

#### Computational complexity

The methods for machine learning used in this study vary in computational complexity. Deep learning models require more computing power during training and inference phases. Convolutional neural network training may be computationally demanding and often requires a bit of GPU time and resources. In contrast, while computationally straightforward, traditional machine learning algorithms like logistic regression and random forests may still be successful when used in combination with appropriate feature engineering and data prior to treatment methods. Batch processing and early stopping circumstances in order to speed up model training and preserve convergence were included in the study. In conclusion it's important to take computational complexity in consideration for the preparation and execution of machine learning experiments.

## Experimental results

The results obtained and the plots made are mentioned to get a broader understanding of the paper. The plots used in the visualisation of the used dataset are further the depiction of the relationships among the parameters in the data. Below are some graphical and tabular illustrations of the results obtained by the modelling. As shown in Table 3 we can see the highest and the lowest range of accuracy and other performance parameters clearly.

**Table 3:** Accuracy Score of ML Models used.

ML model	MAE	MSE	RMSE	Accuracy
Random forest regression	24.25	1522.14	39.01	78.88
XGboost regression	31.73	1998.23	44.70	81.23
Decision tree	23.60	2333.29	48.30	67.42
K-nearest neighbor	26.46	3282.77	57.29	54.17
Linear regression	44.83	3687.54	60.72	48.52
Artificial neural network	47.55	4530.75	67.68	36.05

This is the tabular depiction of the Mean Absolute Error (MAE), Mean Square Error (MSE), Root Mean Square Error (RMSE) and the accuracy of various ML models after the compilation of the code to obtain the desired results.

**Figure 2:**
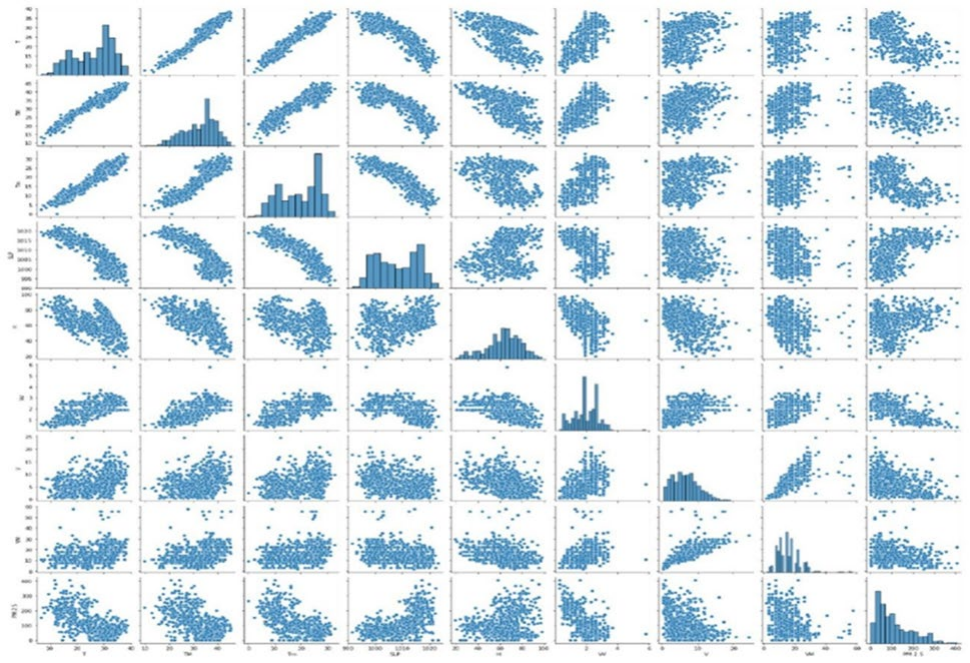
sns pair plot for all the data fields in the provided dataset.

The pair plots explained in the Fig. 2 is basically the depiction of the correlation of each parameter in the dataset to the other. These graphs predict the relationship parameters between all the functional parameters that are present on the dataset.

The data fields used here are plotted as pair plot. A pair plot manages to depict the bivariate data frames of the dataset. The diagonal plots are univariant plots in the given figure.

**Figure 3:**
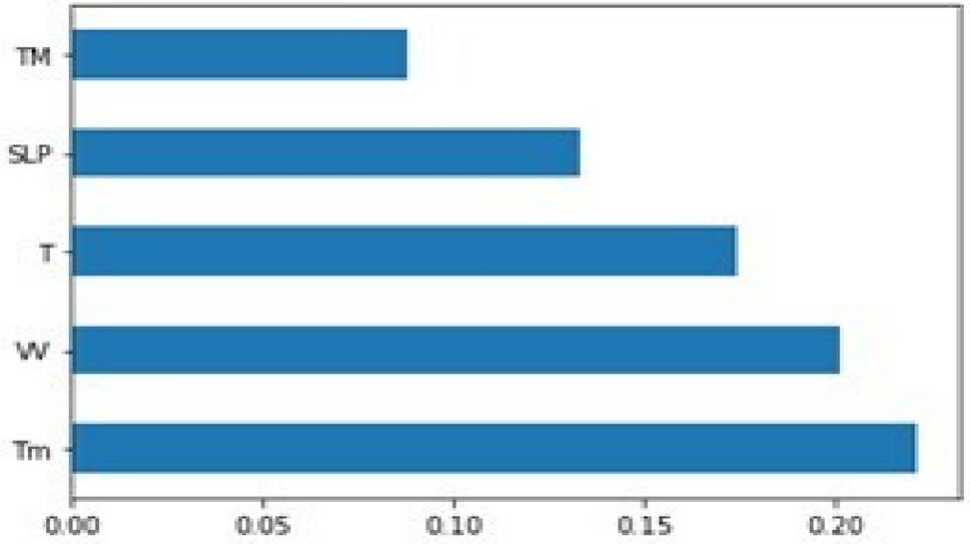
Graph of feature importance for better visualization.

The graph shown in Fig. 3 further depicts the feature importance of all the different parameters present in the data to provide the best feature selection mechanism for the model predictive systems.

This graph shows the visualization of importance of different features present in the dataset after feature scaling.

**Figure 4:**
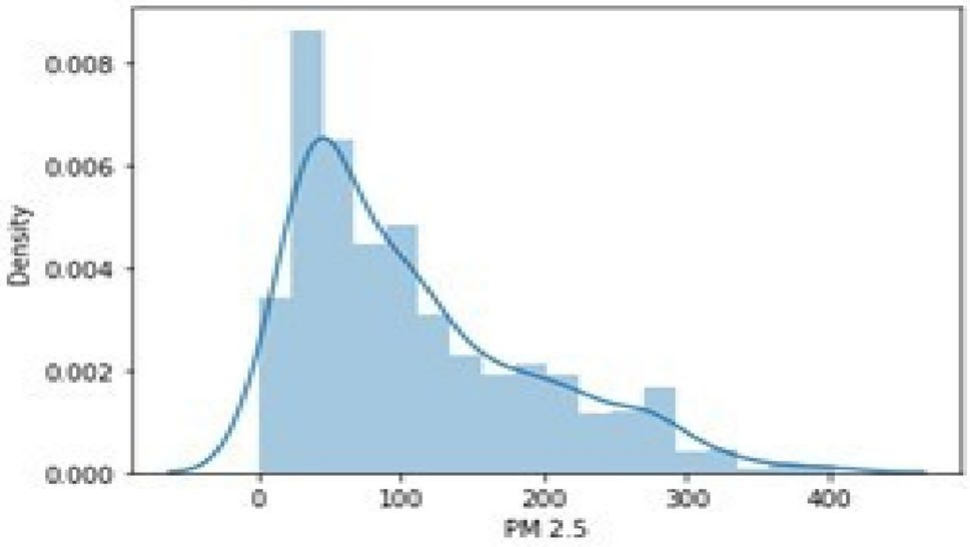
Histogram representing PM2.5 density.

The graph presented in Fig. 4 discusses about the density variation of the PM 2.5 (particulate matter).

The graph is represented of PM2.5 density as per the data available in the dataset.

## Result and discussions

The findings from our study shed light on the complicated relationship between lung cancer incidence rates and the Air Quality Index (AQI) in the discussion section. Though the results we obtained demonstrated a significant positive correlation between high AQI levels and a higher rate of lung cancer, it is essential to recognise the challenges involved in proving causation in an observational study. Our results imply that poor air quality may increase the risk of lung cancer, but other factors, such as smoking habits and occupational exposures, may obscure this relationship. Additional study of putative biological processes by which air pollution may affect lung carcinogenesis is necessary given the complex nature of lung cancer aetiology. However, our findings highlight the importance of initiatives to enhance air quality for public health, demonstrating need for stringent air Inspections of quality and thorough initiatives to prevent lung cancer, especially in regions with high AQI numbers. Further research should look at these procedures and possible remedies to mitigate the impact of poor air quality on lung cancer risk.

The results obtained in the proposed model are discussed on the basis of the performance parameters that have been evaluated and the best accuracy range 81–98% is obtained by XGboost model as compared to the accuracy of 91% by XGboost in research conducted by K. Kumar et al. Also, the second highest range of accuracy is obtained by Random Forest (79 to 97%).

## Conclusion derived

According to the analysis performed the AQI is increasing rapidly which can be determined by using XGboost Regression. AQI major pollutant PM2.5 is directly affecting lung cancer patients by causing microenvironmental alterations in lung cancer leading to increase in inflammation cells also triggering asthma and COPD. As a next step we would like to find more pollutants directly affecting the lung cancer patients and people suffering from respiratory diseases. Further we can test the model using deep learning or hybrid models.

## Future scope

The proposed study can be further extended in the following aspects:

Various genetical mutations(alterations) have been associated to a greater possibility of lung cancer. Some machine learning algorithms can evaluate massive datasets carrying the genetic knowledge for the identification of the mutations. Moreover, it can analyse the possibility of having lung cancer of a specific person. Some machine learning algorithms like deep neural networks can be engaged to determine thorough genetic datasets. Convolutional neural network can spontaneously derive some characteristics from genetic arrangements, determining exquisite mutations connected with lung cancer possibility.

The patient’s medical background, their style of living, and various applicable data to anticipate the possibility of having a cancer can be evaluated by using some machine learning algorithms which can also be used to predict the medication for the patients having severe problems. Machine learning models can be accustomed for analysing individuals’ data, containing medical pictures, medical variables, and microscopic markers, for the estimation of individuals condition and chances of living.

The treatment process which can be the adequate for the particular patient which can give the person a specially made prescription and can advance the health of the patient can be detected by applying some machine learning algorithms. Recurrent neural networks (RNNs) or concentration mechanisms can forecast patient information and genetic description to forecast medication feedbacks.

The chances of a person’s survival can be concluded with the help of these techniques which can be capable to give advice for the improvement of the patient outcome.

## Data Availability

All data used in this manuscript have been presented within the article.
